# HPV Lesions and Other Issues in the Oral Cavity Treatment and Removal without Pain

**DOI:** 10.3390/ijms222011158

**Published:** 2021-10-16

**Authors:** Salima El Moussaoui, Mireia Mallandrich, Núria Garrós, Ana Cristina Calpena, Maria José Rodríguez Lagunas, Francisco Fernández-Campos

**Affiliations:** 1 Departament de Farmàcia, Tecnologia Farmacèutica i Fisicoquímica, Faculty of Pharmacy and Food Sciences, University of Barcelona, Av. Joan XXIII 27-31, 08028 Barcelona, Spain; selmouel9@alumnes.ub.edu (S.E.M.); nuriagarros98@gmail.com (N.G.); anacalpena@ub.edu (A.C.C.); 2 Institut de Nanociència i Nanotecnologia IN2UB, Universitat de Barcelona, 08028 Barcelona, Spain; 3 Departament de Bioquímica i Fisiologia, Faculty of Pharmacy and Food Sciences, University of Barcelona, Av. Joan XXIII 27-31, 08028 Barcelona, Spain; mjrodriguez@ub.edu; 4 Reig-Jofre Laboratories, Av. de les Flors s/n, 08970 Sant Joan Despí, Spain; ffernandez@reigjofre.com

**Keywords:** oral condyloma, Human Papilloma Virus, Ketorolac, pain, inflammation, sodium alginate, hyaluronic acid

## Abstract

Due to different oral and dental conditions, oral mucosa lesions such as those caused by the human papilloma virus and temporomandibular joint pathologies often have to be treated by surgical, ablative or extractive procedures. The treatment and control of pain and inflammation during these procedures is essential to guarantee the patient’s well-being. For the foregoing reason, a hydrogel based on sodium alginate and hyaluronic acid containing 2% of ketorolac tromethamine has been developed. We characterized it physically, mechanically and morphologically. The rheological results suggest that the formulation can be easily and gently applied. Ex vivo permeation studies show that Ketorolac Tromethamine is able to penetrate through the buccal and sublingual mucosae, in addition to being retained in the mucosae’s structure. Through an in vitro test, we were able to evaluate the role that saliva plays in the bioavailability of the drug, observing that more than half of the applied dose is eliminated in an hour. The histological and cytotoxic studies performed on pigs in vivo showed the excellent safety profile of the formulation, as well as its high tolerability. In parallel, a biomimetic artificial membrane (PermeaPad^®^) was evaluated, and it showed a high degree of correlation with the oral and sublingual mucosa.

## 1. Introduction

Various lesions in the oral cavity have been related to the Human Papilloma Virus (HPV) infection: verruca vulgaris (VV), squamous cell papilloma (SP), condyloma acuminatum (CA), and multifocal epithelial hyperplasia (MFEH) [1]. All of them are a benign hyperplastic exophytic proliferation of the oral epithelium [2], caused by different HPV genotypes. Subtypes 6 and 11, with a low-oncogenic risk, are the most commonly found and cause CA in both the oral cavity [2] and in the anogenital region [3]. Labial mucosa, soft palate and lingual frenum are the most common locations of CA [4] and koilocytes can be observed in histopathologic sections [5]. All HPV-related oral lesions present clinical similarities, and therefore, a biopsy is necessary for a precise diagnosis.

Although CA is considered a benign lesion, clinical infections with the high-risk genotypes 16 and 18 have been found to cause oral and genital CA and have been associated with malignant lesions [6]. Spontaneous remission of oral CA is possible [5], but if this is not the case, there are different treatments to eliminate it. Surgical therapy seems to be the preferred treatment over Trichloroacetic acid (TCA), cryotherapy, and CO_2_ laser because these methods often induce artifactual changes that compromise the diagnostic capabilities of the pathologist [1]. Surgical or ablation treatments have the advantage in that the lesion (s) are removed in a single session and, typically, are quick interventions. However, they are procedures that generally require anesthesia and the control of pain and inflammation. The possibility of recurrences should not be ruled out. In addition to lesions caused by HPV, there are a variety of conditions and diseases of the oral cavity requiring surgical, ablative, or extractive interventions that involve mild to severe pain and inflammation, such as certain tumors of the oral mucosa, temporomandibular joint pathologies, facial trauma, and which could require dental interventions, etc. Pain management in minor surgical and ablative treatments does not always attract the attention it deserves, even though it is crucial for patient satisfaction in that they feel well and are mightily encouraged to follow treatment adherence. Surgery and ablative techniques usually require prior local anesthesia, and the postoperative pain and inflammation should also be controlled. For pain management of these processes, analgesics, anesthetics, and anti-inflammatory drugs can be combined in various regimens.

Ketorolac is a non-steroidal anti-inflammatory drug (NSAID) with a potent analgesic effect and a moderate anti-inflammatory action. It is indicated to treat moderate to severe pain. The analgesic ketorolac potency has been equated to that of several opioids [7] without presenting the problems associated with these drugs, such as tolerance or sedation [8]. Its use both pre [9] and postoperatively [10] has been analyzed, showing successful results. Ketorolac is marketed as tromethamine salt and can be administered orally, intramuscularly, intravenously, and by nasal or ophthalmic processes. Several authors have studied the analgesic safety and efficacy of ketorolac tromethamine (KT) after its topical application in different mucoadhesive formulations on the oral mucosa [11,12]. The results were satisfactory and promising. Therefore, we are able to propose its use during the removal of oral condyloma.

Mucoadhesive topical formulations have advantages over the most common routes, such as a simple and painless application and a better bioavailability of the active ingredient, allowing formulations with lower doses and inducing fewer side effects [13]. However, when formulating drugs intended to be applied to the oral mucosa, certain aspects need to be considered and may limit the success of our formula. One of them is the biology and histology of the mucosa. The lining of the oral cavity includes the buccal (cheeks), sublingual, gingival, palatal, and labial mucosa. These are made up of closely compacted epithelial cells, which help fulfill the mucosa’s primary function: to protect the underlying tissues from external agents and fluid loss [14]. The drug to be designed must be able to cross the mucosal barrier. There are different factors to consider, such as the tissue’s permeability, the drug’s molecular weight, the partition coefficient (octanol/water) log P, and all aspects that are related to the formulation: the release capacity of the drug from the vehicle to tissue, pH, and the formulation’s biocompatibility with the target tissue.

In this work, we designed and formulated a 2% ketorolac tromethamine hydrogel composed of sodium alginate as a polymer to be applied to the buccal and sublingual mucosae to treat pain and inflammation before, during, and after surgical, ablative, or extractive procedures. In order to reduce damage to the mucous membranes, hyaluronic acid (HA) was incorporated into the formulation, taking advantage of its well-known and well-documented regenerative and moisturizing action, as well as its role in strengthening cell resistance to mechanical damage [15]. In addition, HA also has gelling properties, which are excellent for the type of formulation to be made. High and low molecular weight HA have been used. The low-molecular-weight HA can penetrate to slightly deeper layers and there it acts regeneratively, while the high-molecular-weight HA acts at a more superficial level. The physicochemical, mechanical, and morphological characteristics of the gel have been analyzed, and the biopharmaceutical properties were examined by ex vivo permeation tests and in vivo administration.

The results show that alginate-HA hydrogel with 2% KT is a promising formulation for combating pain and inflammation, the two common side effects of surgical, ablative, and extractive treatments of the oral cavity.

## 2. Results

### 2.1. Alginate Gel-Ketorolac Characterization

#### 2.1.1. Appearance and pH Evaluation

The finished prepared hydrogel had a translucent light yellowish appearance, considerable consistency, and poor flowability. After allowing it to stand untouched for 24 h, no bubbles or undissolved suspended particles were seen. The pH was 7.2, which is within the normal intraoral pH range (6.8–7.8) [16].

#### 2.1.2. Swelling and Degradation Studies

To determine KT hydrogel’s capacity to incorporate solvent in the matrix, the swelling ratio (SR) was measured. The previously dehydrated KT hydrogel was immersed in PBS, and the weight gain was noted at predetermined time intervals.

**Figure 1:**
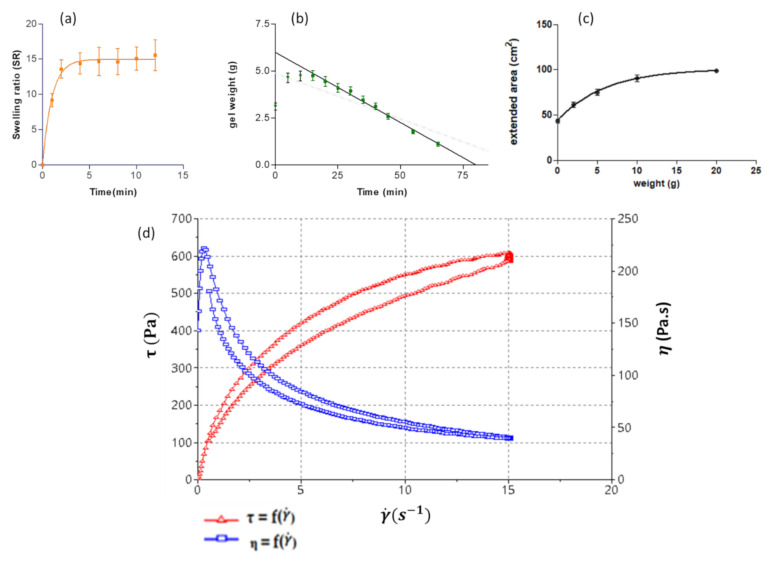
Characterization of the alginate-HA-gel KT 2%: (**a**,**b**) Modelling plots represented as variations in the hydrogel’s weight over time: Swelling Ratio (SR) and degradation assay, respectively. (**c**) Extended area in cm^2^ after applying 2, 5, 10, and 20 g of weight. Expressed as means ± standard deviation (n = 3). (**d**) Flow Curve of alginate-HA hydrogel KT 2%. The blue curve represents the formulation viscosity. The red curve represents the shear stress of the formulation.

As shown in Figure 1a, the KT hydrogel can incorporate up to 15 times its weight in 5 min, showing great hygroscopy. The increase in weight caused by incorporating water in the matrix follows a first kinetic order (one phase association) with a kinetic constant value Kd = 1.012 min^−1^.

The degradation kinetics was determined by immersing the fresh hydrogel in PBS and evaluating the weight changes. In the first minutes, the hydrogel’s weight increases because the polymers are able to include more water in their structure, due to their hygroscopic nature, as previously described. The degradation begins to be appreciated from minute 20 (Figure 1b). The hydrogel degradation followed a zero-order kinetics, with a kinetic constant degradation value (Kd) of 0.075 min^−1^ (r^2^ = 0.993). After about one hour, about 84% of the total hydrogel weight was degraded.

#### 2.1.3. Extensibility

The extensibility was determined by observing the area over which the hydrogel extended after applying specific weights (0, 2, 5, 10, and 20 g). The results are shown in Figure 1c, where it is observed how the formulation extends to cover an area of 98.87 cm^2^. The increase in extension was 51.61 cm^2^. This increase followed a pseudo-first-order association with a constant value of 0.013 g^−1^. These results show how the KT-hydrogel has good extensibility for the treatment’s target condylomatous lesions.

#### 2.1.4. Rheological Profile

To carry out an adequate characterization of semi-solid products, it is essential to evaluate rheological behavior. In the oscillatory study, a strain sweep of 0 to 500 Pa, at 1 Hz, was first performed to determine the linear viscoelastic zone. This said linearity was observed to be between 0.4 and 100 Pa. To perform the frequency sweep, which allows how the product behaves at low and high frequencies to be determined, the shear is set to a value within the linear viscoelastic zone, in our case 10 Pa. Initially, at low frequency, the viscous mode predominates up to the value of 0.8 Hz; from there, the elastic mode predominates. At 0.7902 Hz, the two modules G’ = G” = 329.2 Pa (crossover point) are equalized.

Figure 1d shows the flow curves. It can be seen that the shear stress does not increase linearly with the shear rate, so it is concluded that the fluid is non Newtonian pseudoplastic. To confirm the rheological profile, the experimental data were fitted to different equations (Table A1).

The model that best fits is the Cross equation in both up and down curves, which usually explain the behavior of pseudoplastic material in a broad range of shear rates. Under certain assumptions, the Cross equation could be simplified to the power law equation [17]. This is the reason why in the ramp-down curve, the regression coefficient of the Cross equation is the same as the Herschel-Bulkley equation. In fact, in the ramp-up curve, the regression coefficient of Herschel-Bulkley exhibits a good fit, and considering that the Cross equation has four parameters, in contrast with the three parameters of the Herschel-Bulkley equation, the latter could be more appropriate to describe the flow behavior (avoiding an overparameterization) of the produced hydrogel. Nevertheless, both equations confirmed that the formulation exhibits a pseudoplastic flow (exponent n < 1) with yield stress around 150 Pa.s.

As shown in Figure 1d, the flow curve presents a hysteresis loop, which denoted a thixotropic behavior or a viscosity time-dependence, indicating that the microstructure of the gel is altered as it was sheared with a disturbance degree of Δa = 1582 Pa/s. The medium viscosity determined at 15 s^−1^ is 39.61 ± 0.44 Pa.s.

#### 2.1.5. Morphological Study

**Figure 2:**
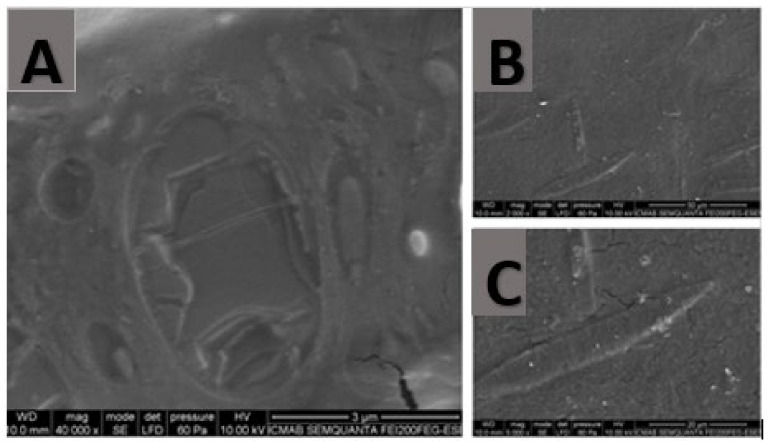
SEM images of sodium alginate and hyaluronic acid gel with 2% KT. (**A**) Magnification 40.000×, scale bar 3 µm; (**B**) magnification 2.000×, scale bar 50 µm; (**C**) magnification 5.000×, scale bar 20 µm.

To determine the hydrogel morphology, the formulation was analyzed by Scanning Electron Microscopy (SEM). In SEM, an electron beam with low energy is radiated onto the material and scans the sample’s surface. It can be seen that the hydrogel has a very compact structure, and no pores are observed in its microstructure. In the 40.000× magnification (Figure 2), small cavities are seen, in which the KT in solution could be lodged.

### 2.2. In Vitro Release Assay on Membranes and Biomimetic Membranes

The hydrogel’s ability to release the KT was evaluated using Franz-type cells according to the reported methodologies [18]. In a previous study, two synthetic membranes, nylon and polyethersulfone (PES), were evaluated to select the membrane that allows a greater release of a free drug solution (at the same KT concentration as the hydrogel). Nylon was discarded for presenting major resistance to the release of KT (data not shown). Thus, the in vitro release assay of the alginate-HA 2% KT was conducted with the PES membrane and the synthetic biomimetic membrane.

**Figure 3:**
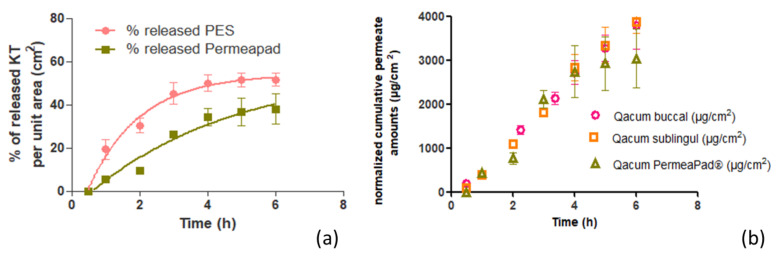
In vitro and ex vivo assays. (**a**) Representation of the percentage of released KT per unit area (cm^2^) from the PES membrane (pink curve) and the PermeaPad^®^ biomimetic membrane (green curve). (**b**) Cumulative amount of KT permeated (μg/cm^2^) under an infinite dose regimen through buccal and sublingual mucosae and biomimetic membrane upon application of KT hydrogel. Values represent means ± SD (n = 3).

Figure 3a shows the release profile of KT from the hydrogel in the PES membrane and in a synthetic biomimetic membrane, called PermeaPad^®^, which is claimed to mimic the permeability behavior of oral mucosa [19]. The membrane is composed of two cellulose membranes, and there is one lipidic barrier between them.

After the assay, the results show that the hydrogel can release up to 4.12 mg/cm^2^ of KT through the PES membrane, corresponding to 51.6% of the total dose seeded (8.00 mg) per unit area, which was achieved within 6 h, showing a release rate of 0.66 h^−1^. At the same time, regarding the results obtained with the biomimetic membrane, 38.0% of KT was released through this membrane, which corresponds to a total amount of 3.04 mg/cm^2^. This amount was achieved in 6 h with a release constant of 0.23 h^−1^. The differences were statistically significant (*p*-value for *t*-test of 0.001).

The release curves were adjusted to different kinetics showing the best fit and correlation for first-order kinetics (see r^2^ values in Table 1).

### 2.3. Ex Vivo Transmucosal Permeation Assay

The KT permeations across porcine buccal and sublingual mucosae were performed ex vivo according to previously reported methodologies [20] under an infinite-dose regimen [21]. Figure 3b shows the permeation profiles of KT from the alginate-HA gel through the buccal and sublingual mucosae as well as through the biomimetic membrane.

The results between the two mucosae, buccal and sublingual, were compared by a *t*-test statistical analysis. The permeated amounts through the buccal and sublingual mucosae after 6 h exposure to the KT hydrogel were very similar: 3790.61 µg/cm^2^ of KT (47.4% of the total dose applied) in the case of the buccal mucosa and 3864.93 µg/cm^2^ (48.3%) within the sublingual mucosa (*p* value = 0.836). The permeability coefficients (Kp) were also very similar: 0.052 cm/h and 0.056 cm/h, respectively (*p* = 0.183). The same happens with the other calculated biopharmaceutical parameters (see values in Table 2). The transmucosal flow values (Jss) obtained were high: 666 µg/h·cm^2^ and 714.59 µg/h·cm^2^, respectively (*p* = 0.192). Said values are in agreement with the release test results, in which a great capacity of KT to be released from the hydrogel formulation in which it had been dissolved was observed. The latency time was low in both tissues. In the buccal mucosa, the steady state is reached quickly, after the first 8 min (0.13 h) of the test and in the sublingual mucosa after approximately 23 min (0.38 h) (*p* = 0.004). The estimated steady-state plasma concentration (Css) was calculated considering a hypothetical area of application of 5 cm^2^, the human plasma clearance of ketorolac (1840 mL/h) [22] for an individual with a mean weight of 80 kg. For the buccal mucosa, the estimated Css was 1.13 µg/mL, and for the sublingual mucosa, it was 1.21 µg/mL (*p* = 0.219). These values are within the therapeutic range of 0.3 to 5 µg/mL of KT [23], so the studied formulation would allow sufficient transmucosal permeability to achieve systemic therapeutic concentrations. The extraction study determines the amount of KT that the tissues subjected to the permeation test are capable of retaining. Both mucous tissues can retain a certain amount of KT. The buccal mucosa was able to retain a total of 127.34 µg/cm^2^ of KT and the sublingual mucosa 150.97 µg/cm^2^. The statistical study (*t*-test) gave a *p*-value greater than 0.05 (0.377), so we can conclude that both mucosae have the same KT retention capacity.

The differences in the results were not statistically significant, except for the Tlag values. Therefore, it can be concluded that the transmucosal permeation of KT through both mucosae, buccal and sublingual, are the same, only that through the buccal mucosa, the therapeutic effect would be achieved more rapidly.

The cumulative amount of ketorolac permeated through each tissue over a long time is shown in Figure 3, and the permeation parameters are calculated in Table 2.

#### In Vitro—Ex Vivo Correlation

**Figure 4:**
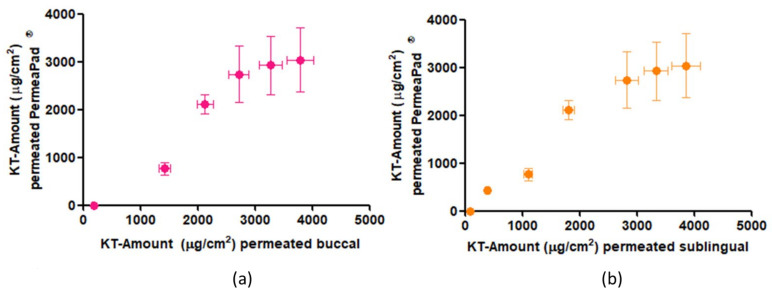
In vitro–ex vivo correlation: (**a**) correlation of in vitro permeation through PermeaPad^®^ membrane and ex vivo permeation through buccal mucosa, (**b**) correlation of in vitro permeation through the PermeaPad^®^ membrane and ex vivo permeation through sublingual mucosa.

In order to analyze the biocompatibility of the PermeaPad^®^ synthetic membrane with the mucosa of the oral cavity, the correlation of the permeation results through the buccal and sublingual mucosae with the results of the release test through the biomimetic membrane (Figure 4a,b) was calculated. The regression coefficient (r^2^) was 0.94 between PermeaPad^®^ and buccal mucosa and 0.95 between the PermeaPad^®^ membrane and sublingual mucosa, showing a strong positive correlation.

Figure 3b shows the profiles of the permeation kinetics through the ex vivo tested mucosa and the PermeaPad^®^ biomimetic membrane. It can be seen how the permeation kinetics through the mucosa and the PermeaPad^®^ membrane are the same for up to 4 h. In the last 2 h, it seems as if a lesser amount had been permeating through the PermeaPad^®^ membrane. Therefore, a correlation of the results was made at up to 4 h to see if it gave a better result than the correlation at 6 h. The r^2^ at 4 h was 0.95 between the PermeaPad^®^ membrane and buccal mucosa and 0.96 between the PermeaPad^®^ and sublingual mucosa. As can be observed, the values of the correlation coefficients at 4 and 6 h practically do not differ practically; therefore, it can be concluded that the PermeaPad^®^ biomimetic membrane can predict the permeation of alginate-HA-KT gel through the buccal and sublingual mucosa, for at least 6 h, with an excellent correlation.

In addition to the correlation study, a statistical study (one-way ANOVA) was performed between permeation parameters. The results were a *p*-value greater than 0.05 for all parameters except for the retained amounts of KT in the tissues and biomimetic membrane and the Tlag (Table 2). Besides the ANOVA test, Tukey’s multiple comparison test was carried out to determine which parameters differed. The results were that the biomimetic membrane reached the steady state just as quickly as the buccal mucosa (in about 10 min) but faster as the sublingual mucosa (about 23 min) and that the two mucosae were able to retain more than twice as much KT as the PermeaPad^®^ membrane.

Taking into account all these results, it can be concluded that the PermeaPad^®^ membrane is able to predict with a high correlation the permeation kinetics of KT both in buccal and sublingual mucosae, and it has a significantly lower drug retention capacity.

### 2.4. In Vitro Study of the Influence of Saliva on Drug Elimination

**Figure 5:**
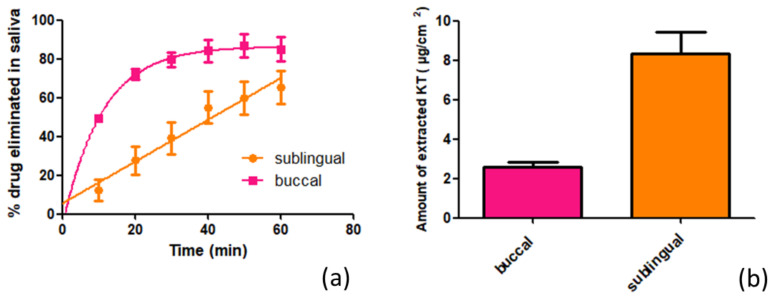
In vitro study of influence of saliva on drug elimination: (**a**) percentage of KT eliminated by the action of artificial saliva at a flow rate of 0.24 mL/min in buccal (pink curve) and sublingual (orange curve) mucosa as a function of time; (**b**) Amounts of KT extracted from the mucosa (buccal—pink; sublingual—orange) after the in vitro study. Results expressed as mean and SD (n = 3).

The in vitro simulation aims to determine the amount of KT that would be eliminated from the mucosa and would end up being swallowed under the influence of artificial saliva exposed to the mucosa by a peristaltic pump. Salivary flow is highly variable and depends on many factors and stimuli. Any value above 0.10 mL/min is considered acceptable, and the average is around 0.30 mL/min [24]. Taking into account the capacity of the available peristaltic pump, a continuous flow of 0.24 mL/min was established to perform the in vitro simulation. The study lasted 1 h, and samples of the saliva dropped down were extracted every 10 min. In Figure 5a, the elimination profile of KT is represented as a function of time.

It is observed how the elimination through the buccal mucosa follows first-order kinetics (r^2^ = 0.92), and through the sublingual mucosa, it follows zero-order kinetics (r^2^ = 0.96). This differential behavior in the kinetics dissolution of the drug in the saliva is striking. The total amounts eliminated and therefore swallowed by the action of saliva after one hour of the hydrogel application were 2239.88 µg/cm^2^, equivalent to 85.1% of the total KT applied (4656 µg), in the buccal mucosa, and 1931.01 µg/cm^2^, equivalent to 65.2% of the total KT (5230 µg), in the case of the sublingual mucosa (*p* = 0.008). The results of the extraction of KT retained in the mucosa are represented in Figure 5b, in which it is seen that the sublingual mucosa is capable of retaining 8.34 µg/cm^2^ of KT (2.01% of the total amount applied per unit area) and the buccal mucosa 2.58 µg/cm^2^ (corresponding to 0.62% of the total amount applied per unit area), with a *p*-value of 0.006, which means that the sublingual mucosa is capable of retaining twice as much KT as the buccal mucosa.

### 2.5. In Vivo Study in Pigs and Histological Study

After the encouraging results obtained in the in vitro and ex vivo studies, we wanted to analyze the behavior of the hydrogel under the effect of salivation in live pigs. The ex vivo permeation study was carried out under an infinite dose regimen to determine the permeation capacity of the oral mucosae. In this case, to better analyze what the real application of the hydrogel would be like, a finite dose regimen was chosen. The dose applied was 5 mg/cm^2^ of formulation, as recommended by the Organization for Economic Cooperation and Development (OECD) [25]. The test duration was established at two hours for the in vitro test results on the influence of saliva, where it was observed that after the first 60 min, a large part of the applied hydrogel had been eliminated by the action of saliva. Another concern was to avoid stress on the pigs, and thus not to lengthen the in vivo test with animals unnecessarily.

**Figure 6:**
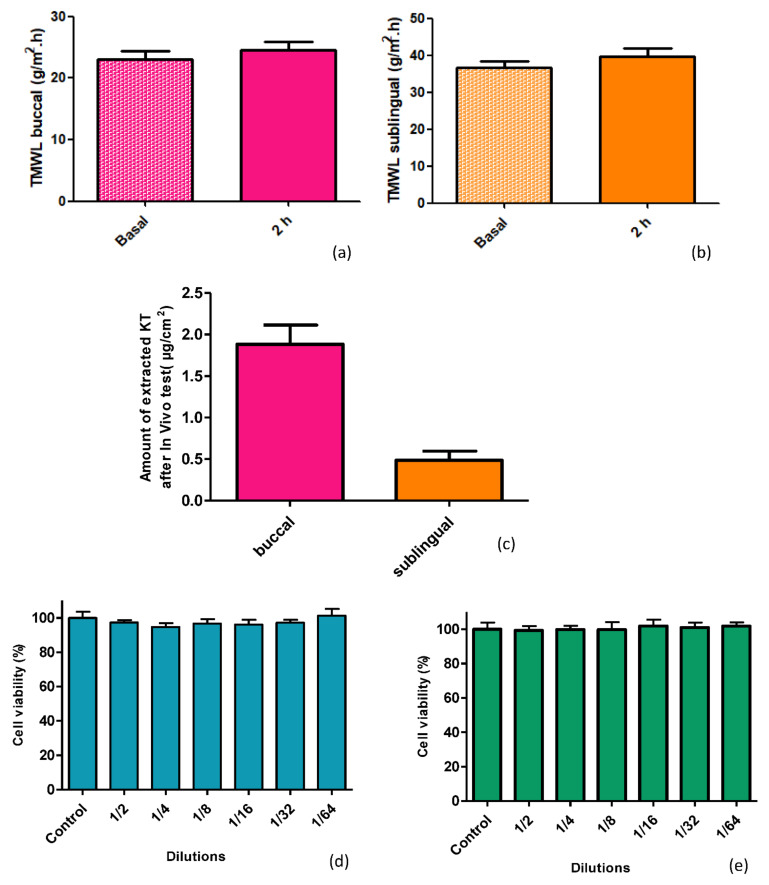
In vivo studies: Integrity of the buccal (**a**) and sublingual (**b**) mucosae. TMWL values taken before the alginate-HA 2% KT gel application (basal) and 2 h after application. (**c**) Amount of extracted KT (µg/cm^2^) from the mucosae after in vivo test on pigs. Results represented as mean and SD (n = 3). (**d**,**e**) Cell viability (%) after the MTT test for different dilutions of the 2% KT alginate-HA gel and the KT-solution, respectively. Control: untreated cells in their environment. Results expressed as mean and SD (n = 8).

Figure 6a,b shows the TMWL values for the buccal and sublingual mucosae before applying the hydrogel (basal) and at 2 h post-application. There were no statistically significant differences (*p* < 0.05) between post-application and basal TMWL values, indicating that the 2% KT formulation did not cause the de-structuring of the mucosal epithelium.

Then, before euthanizing the animals, a blood sample was taken to assess whether systemic drug levels could be reached, as predicted in the ex vivo permeation study. Concentrations between 0.3 and 5 µg/mL of KT are considered concentrations within the therapeutic range [23]. After analysis by HPLC, no peak of KT was detected (quantification limit for KT = 0.011 µg/mL). The buccal and sublingual mucous membranes were extracted once the animals had been sacrificed to determine the amount of KT that could have been retained. As can be seen in Figure 6c, the buccal mucosa can retain 1.88 µg/cm^2^ of KT, and the sublingual mucosa 0.48 µg/cm^2^. Differences were statistically significant (*p* = 0.0007).

#### Histological Analysis

**Figure 7:**
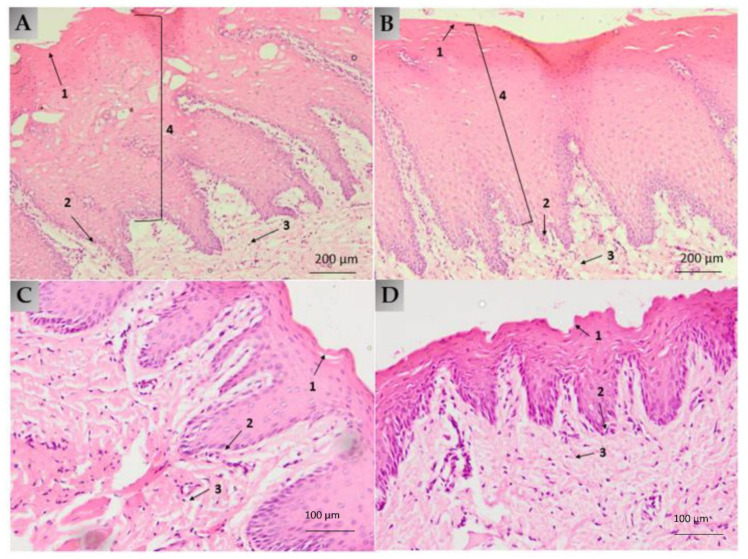
(**A**) Buccal mucosa histology under basal conditions, without gel (100× magnification); (**B**) buccal mucosa histology after 2 h gel exposure (100× magnification); (**C**) sublingual mucosa histology under basal conditions, without gel (200× magnification); (**D**) sublingual mucosa histology after 2 h gel exposure (200× magnification). (1) Keratinized or non-keratinized stratified squamous epithelium; (2) basal layer; (3) lamina propria; (4) dermal papilla.

The histological study was carried out with two of the four pigs used for the in vivo test, extracting the buccal and sublingual mucosa, and analyzing the histology of the tissues in the microscope before and after the application of the alginate-HA gel. As can be seen in Figure 7, both mucous membranes do not present differences in their histology before and after the application of the gel. No severe cytopathic effects (alterations in cell morphology or epithelium structure) were found in treated samples (Figure 7b,d), which demonstrate that the transmucosal application of the alginate-HA and KT gel is safe and well tolerated by the target tissues.

### 2.6. Cytotoxicity

We examined the impact of alginate gel on the cell growth of the intestinal epithelial cell line Caco-2. For this, Caco-2 cells were exposed to different alginate-HA gel dilutions. The 2% KT alginate-HA gel was diluted in DMEM medium, and dilutions containing ketorolac ranging from 0.715 to 0.09 µg/mL were used in this analysis of cytotoxicity. Living cell numbers were calculated by the MTT assay carried after 24 h incubation. Results showed that cell viability was not altered by the alginate-HA gel, at the concentrations tested (Figure 6d). The viability of cells treated with a ketorolac drug solution used as a control (Figure 6e). In both cases, the viability profile was similar (*p* > 0.05).

## 3. Discussion

We elaborated a 2% ketorolac tromethamine hydrogel composed of sodium alginate as a polymer to be applied to the buccal and sublingual mucosae with the aim of treating pain and inflammation before, during, and after surgical, ablative, or extractive procedures in the oral cavity. The hyaluronic acid was incorporated into the formulation because of its well-known regenerative, moisturizing and strengthening properties [15]. Both, low and high molecular weight hyaluronic acids have been used. The low-molecular-weight HA can penetrate to slightly deeper layers, and there, it can act regeneratively, while the high-molecular-weight HA acts at a more superficial level [26].

The physicochemical, mechanical, and morphological characteristics of the gel have been analyzed. The pH was within the normal intraoral pH range (6.8–7.8) [16], thus no disruptions, neither the biota nor the functions of saliva in the oral cavity, are expected. The alginate-HA gel showed to be hygroscopic in nature since it is able to uptake 15-fold its weigh in solvent, and their components can disperse in the medium relatively quickly compared to other polymer-based hydrogels. Mallandrich et al. studied the degradation of a 2% carbopol hydrogel, which required 24 h to be thoroughly degraded [27].

When formulating gels, determining the extensibility is crucial to ensure that the formulation is pleasant to use and has a comfortable application. That is why very high (very fluid) or very low (very viscous) extensibility should be avoided. The patient’s compliance will be affected by the sensory feeling of the formulation. Inoue and co-workers investigated the correlation between the physical properties of different formulations and the sensory feeling [28]. This means that rheological studies are essential to evaluate the galenic features and the suitability of a formulation. Despite the compact structure of the alginate-HA 2% KT gel observed by SEM, the gel exhibited good extensibility and an ideal rheological behavior for the indication of the hydrogel. Pseudoplastic behavior is interesting because it allows a smooth and easy extension application by dabbing without high pressure and, therefore, painlessly. Furthermore, thixotropy also displays interesting behavior in semi-solid products because the formulation’s change in structure results in fluidization that facilitates the product’s application. This is an interesting outcome since the mucous membranes are already sensitive tissues per se and even more so after a surgical, ablative, or extractive intervention.

The biomimetic membrane PermeaPad^®^ was tested and compared to the buccal and sublingual mucosae. It was observed that the biomimetic membrane correlated well with both mucosae. These results are in agreement with other researchers’ work. Bibi et al. [19] investigated the use of PermeaPad^®^ as a predictor in the buccal absorption of Metoprolol solution. The authors compared the apparent permeability obtained with PermeaPad^®^ to the previous works performed by other authors, which evaluated the apparent permeability of metroprolol solution in cell culture, in ex porcine buccal mucosa and, finally, in in vivo studies conducted on minipigs. Bibi et al. found good in vitro–in vivo correlation between PermeaPad^®^ and all the three systems evaluated.

Ketorolac tromethamine rapidly diffuses across the mucous membranes of the oral cavity, especially through the buccal mucosa. Under infinite doses and an exposure time of 6 h, ketorolac tromethamine would achieve therapeutic plasma concentrations. Nevertheless, the impact of saliva on drug elimination should not be disregarded, since the main drawback in buccal delivery is that the patient may swallow part of the applied dose before the drug is absorbed, even if it has been released [29]. The in vitro test showed that saliva dragged more than 60% of Ketorolac in 1 h, which is swallowed and follows on from an oral intake. Even despite the saliva’s effect, Ketorolac tromethamine was able to penetrate both mucosae. The alginate-HA-hydrogel was formulated with Ketorolac in the tromethamine salt since this is more hydrophilic and allows it to be better integrated into the hydrogel. However, it is to be expected that once the gel is applied to the mucous membranes, the KT changes and, due to the environment in which it is found, protonates and/or ionizes and that, during the process, the different forms coexist until reaching a balance. These changes will affect the physicochemical properties of the drug, such as its solubility in saliva or the value of the log P partition coefficient, thus modifying its tissue affinity [18,30]. Given that the alginate-HA-hydrogel was formulated with the tromethamine salt, it is likely that this was the predominant form at the beginning of the study when applying the gel on the mucosa and therefore that the process of dissolving KT in saliva was favored. It should not be forgotten, however, that the KT of the formulation is simultaneously being absorbed through the mucosa. This absorption depends on different factors. On one hand, the mucosa histology, and on the other, the KT physicochemical properties. Taking into account that both the buccal and sublingual mucosae consist of a non-keratinized stratified squamous epithelium and that the main difference is the thickness of the said epithelium (the sublingual being between 100 and 200 μm, 8–12 cells thick, and between 500 and 800 μm, 40–50 cells thick, the buccal [24]), it is logical to think that the sublingual mucosa presents less resistance to the passage of KT. From the beginning, the amount of KT that is eliminated by the action of the saliva when the gel is applied on this mucosa is lower compared to when the gel is applied on the buccal mucosa. The kinetic dissolution profile is the result of the sum of these two processes. Through the buccal mucosa, it is observed how at the beginning, the KT dissolution in saliva predominates until after about 30 min, when a balance is reached between what is dissolved and what is absorbed by the mucosa. This behavior adjusts to first-order kinetics. On the other hand, in the sublingual mucosa, by presenting less resistance to the passage of KT through its structure, the dissolution and absorption processes are balanced from the beginning, and this therefore describes zero-order dissolution kinetics. These results show how, apart from the drug physicochemical properties and the mucosa physiological characteristics, other factors such as salivation play a significant role in the bioavailability of KT since, in both mucosae, much more than half of the applied dose was eliminated by the action of saliva, which can be explained, in part, by the high-water solubility of KT, which is eliminated from the mucosa by the action of saliva and ends up being ingested. It is therefore considered that the KT is administered orally. In contrast, the amount that is retained in the mucosa is responsible for the local analgesic and anti-inflammatory action.

Thus, it is observed that the study in live animals and under a finite dose regimen does not reach systemic concentrations of KT. Besides the lower dose, this is probably due to the effect of saliva, as demonstrated in the in vitro study, which can influence by reducing the time that the alginate-HA gel is in contact with the mucous membranes and consequently with the amount of KT that could be permeated. It should be noted that the present study was carried out by administering a single drug dose in order to minimize the test time as much as possible and, therefore, the stress that could be caused to the animals. Other studies applying the alginate-HA gel in a multiple-dose regimen are necessary to analyze the behavior of the hydrogel more accurately in terms of bioavailability and permeability through the mucous membranes of the oral cavity.

When mucosae are damaged, their barrier functions are impaired, resulting in higher water loss [31]. This water loss can be measured by the transmucosal water loss (TMWL) method, which is well established in dermatology and used to assess the integrity of the mucosa barrier in vivo [32]. In the TMWL measurement, the water density gradient that evaporates through the tissue is indirectly measured by placing the measuring device perpendicular to the site of interest and reaching a stable TMWL reading in about 60 s. Before exposing the mucosa to alginate-HA hydrogel, the basal TMWL value was measured. The formulation was then applied to the mucosae, and after 2 h, the TMWL value was measured again. The TMWL values obtained, both basal and 2 h post-application, (around 30 g/m^2^·h for the buccal mucosa and around 40 g/m^2^·h for the sublingual mucosa) show the excellent condition of the mucosa since both have values close to 30 g/m^2^·h, which is considered acceptable for the integrity of the oral mucosa [32]. Thus, the alginate-HA 2% KT does not cause mucosae disruption, being well-tolerated by the target tissues: the histological analysis revealed no differences between the treated and the untreated mucosae. Additionally, the cell viability showed that the alginate-HA 2% KT does not cause cytotoxicity in Caco-2 cells.

## 4. Materials and Methods

### 4.1. Materials

#### 4.1.1. Reagents

Sodium alginate was purchased from Fagron Iberica (Terrassa, Spain). Ketorolac tromethamine and Nipagin were obtained from Sigma-Aldrich (Barcelona, Spain). Nipasol was acquired from Acofarma (Barcelona, Spain), Hyaluronic acids were obtained from Fagron Iberica (Terrassa, Spain); Na2HPO4 and KH2PO4 were supplied by Panreac (Barcelona, Spain), NaCl and KCl were from Merck (Darmstadt, Germany), CaCl from Ferosa (Spain) Hepes was obtained from Fagron Iberica (Terrassa, Spain) and glucose from Sigma-Aldrich (Barcelona, Spain). The Millipore Express^®^ PLUS 0.45 µm PES Membrane was from Merck (Darmstadt, Germany), the Nylon membrane Filter with 0.45 µm pore size from Teknokroma (Barcelona, Spain) and the biomimetic membrane PermeaPad^®^ from InnoME GmbH (Espelkamp, Germany).

The purified water was obtained from a Milli-Q1 Gradient A10 system apparatus (Millipore Iberica S.A.U., Madrid, Spain). All the other chemicals and reagents used in the study were of analytical grade.

#### 4.1.2. Tissues for Ex Vivo Assays

The Bellvitge animal facility services provided the buccal and sublingual mucosae (Landrace Large White race). The Ethics Committee of Animal Experimentation of the University of Barcelona approved the Study Protocol (approved on 10 January 2019). A thickness of 500 µm was dermatomized (GA630, Aesculap, Tuttlingen, Germany) to carry out the test.

### 4.2. Preparation of the Sodium Alginate and Hyaluronic Acid Hydrogel

Drug-loaded hydrogel was prepared at laboratory scale with a KT concentration of 2% *w*/*v*. A concentration of 0.5% *w*/*v* of high molecular and 0.2% low molecular weight HA was used. Sodium alginate concentration was established at 4% *w*/*v*. Firstly, preservatives (nipagin and nipasol at 0.05% and 0.02%, respectively) and KT were added to purified water under continuous stirring. Separately, both HA were dispersed in ethanol (corresponding to a 5% of the final composition); then, the KT-preservatives solution was poured into the HA mixture, and finally, alginate was added gradually to avoid the formation of lumps. Then, the formulation was allowed to rest for 24 h at room temperature.

### 4.3. Gel Characterization

The alginate gel containing 2% ketorolac was characterized in terms of appearance, pH, extensibility, swelling and degradation ratio and rheological behavior.

#### 4.3.1. Swelling and Degradation Tests

The swelling and degradation tests were carried out according to the methodology described by S. El Moussaoui et al. [18]. The swelling test evaluates the hydrogel’s capacity to absorb water within its structure. First, the hydrogels were dehydrated at 40 °C until constant weight. The dehydrated hydrogels were immersed in PBS (pH 7.4) at room temperature for 14 min. The hydrogels were removed from the PBS at predetermined intervals (every 2 min). The excess PBS was removed, and the amount of captured PBS was weighed. The experiment was performed in triplicate. The swelling ratio (SR) was calculated using the following equation:

(1) $$SR=\frac{W_{s}-W_{d}}{W_{d}}$$

where *W_d_* is the weight of the dried hydrogel, and *W_s_* is the weight of the swollen hydrogel at different times.

The degradation test aims to monitor weight loss (WL) as a function of time. The WL was calculated by incubating known amounts of fresh hydrogel (3.017 g) in PBS (pH = 7.4) at 37 °C for 115 min. Three replicates of hydrogel were removed, blotted, and weighed at the following times: 0, 5, 10, 15, 20, 25, 30, 35, 40, 45, 55, 65, 75, 85, 100, and 115 min. The weight loss was expressed as the percentage of weight loss concerning the freshly prepared hydrogel. It was calculated based on Equation (2):

(2) $$WL\left(\%\right)=\frac{W_{i}-W_{d}}{W_{i}}\;100\%$$

where *W_i_* is the initial weight of hydrogel and *W_d_* the weight of hydrogel at different times.

#### 4.3.2. Extensibility

The extensibility was determined in triplicate at room temperature. The formulation was placed between two crystal platforms. Several weights (0, 2, 5, 10, and 20 g) were placed on the top platform for 2 min. The diameters (cm^2^) of the circles which spread out were measured and recorded. The extensibility was calculated from the equation:

(3) Ext=π·d−^24

where *Ext* = extensibility; *d* = average diameter of the extended formulation.

#### 4.3.3. Rheological Profile

The rheological properties of the Alginate-HA hydrogel containing 2% KT were determined by a rotational Haake RheoStress 1 rheometer (Thermo Fisher Scientific, Karlsruhe, Germany) equipped with cone-plate geometry (Haake C60/2° Ti, 60 mm diameter, 0.105 mm gap between cone-plate). Measurements were performed in duplicate at 25 °C (Thermo Haake Phoenix II + Haake C25P). An oscillatory study was carried out, for which a stress sweep of 0 to 500 Pa, at 1 Hz, was first performed to determine the linear viscolastic zone. For the following test, the shear was set at a value between 0.4 and 100 Pa (linear zone), and 10 Pa was chosen to perform the Frequency Sweep test, which allows how the product behaves at low and high frequencies to be determined and recorded.

For the rotational study, the program was adjusted to the following conditions: ramp-up from 0 to 15.0 s^−1^ for 3 min, constant shear rate at 15.0 s^−1^ for 1 min, and ramp-down from 15.0 to 0 s^−1^ for 3 min. The flow data obtained were fitted to different mathematical models (Table A2) to describe the flow curve and characterize the flow properties.

#### 4.3.4. Morphological Study

In order to examine the hydrogel structure, Scanning Electron Microscopy (SEM) was carried out in a JSM-7100F (JEOL Inc., Peabody, MA, USA). The sample was coated with a thin layer of carbon in an Emitech K950 coater (Quorum Technologies Ltd., Kent, UK).

### 4.4. In Vitro Release Assay on Membranes and Biomimetic Membranes

The in vitro release profile was assessed by vertical Franz diffusion cells (FDC 400, Crown Glass, Somerville, NY, USA) with an active diffusion area of 1.77 cm^2^. Hank’s solution pH (7.02) was used as receptor fluid, which was thermostated at 37 ± 1 °C. The stirring rate was set at 500 rpm, and sink conditions were held throughout the experiments. A total of 400 mg ± 10 mg of KT hydrogel was accurately applied to the membranes.

The membranes used were PermeaPad^®^, and polyethersulfone (PES)—the selection of PES membrane was based on a previous study in which a solution of Ketorolac tromethamine was tested through Nylon and PES membranes. Samples of 300 μL from the receiver compartment were extracted over pre-established times (0.5, 1, 2, 3, 4, 5, and 6 h) and replaced with an equal volume of fresh solution. The KT content was analyzed by a validated HPLC method, described in Section 4.8.

The experimental data (cumulative amount of KT per cm^2^) were fitted to different mathematical models (zero and first-order kinetics) so as to choose the best fitting model according to the correlation coefficient (r^2^) value.

### 4.5. Ex Vivo Transmucosal Permeation Assay

The ex vivo permeation tests were conducted on porcine oral mucosa from the pigs’ cheeks and sublingual tissues to evaluate the ability of KT to permeate through the mucosal membranes. The mucosae were dermatomed at a thickness of 500 µm.

The assays were performed on Franz diffusion cells with a diffusional area of 0.64 cm^2^. They were conducted in the same conditions as in the release assay (Section 4.4), adjusting the sampling times to 1, 2, 3, 4, 5, and 6 h. Samples were analyzed with the same HPLC method. From the permeated amounts analyzed by HPLC, the permeation parameters were calculated according to the equations described in Appendix A (Table A3).

#### 4.5.1. Amount of Ketorolac Retained in the Mucosa

Once the permeation assay was finished, the KT retained in the mucosa was extracted. To do this effectively, the residual hydrogel on the mucosa was removed with a swab and cleaned with a gauze soaked in 0.05% solution of sodium lauryl sulfate and rinsed three times with distilled water. The permeation area of the mucosa was cut, weighed, perforated by a thin needle, and incubated with 1 mL of methanol:water (1:1) solution and sonicated for 20 min. The supernatants were analyzed by the HPLC method.

#### 4.5.2. In Vitro—Ex Vivo Correlation

To determine if the PermeaPad^®^ biomimetic membrane can be comparable to the buccal and sublingual mucosae, the ex vivo permeation results and parameters were compared with the release test results performed on the PermeaPad^®^ membrane, and to achieve this, a one-way ANOVA statistical study was carried out.

Furthermore, the correlation between the amounts of KT permeated through PermeaPad^®^ vs. through buccal and sublingual mucosae at each time was calculated with the help of GraphPad software.

### 4.6. In Vitro Study of the Influence of Saliva on Drug Elimination

To simulate the influence of saliva on the KT elimination once the hydrogel had been applied to the mucosa of the oral cavity, the buccal and sublingual mucosae were cut to an area of 2.83 cm^2^ and placed vertically on a support point. A certain amount of KT hydrogel was carefully placed on the mucosa. Using a peristaltic pump, artificial saliva (Table A4) was passed over the mucosa at a 0.24 mL/min flow rate. The study lasted 1 h, taking samples of the artificial saliva that dropped every 10 min. The collected samples were analyzed by HPLC to determine the amount of KT that had been eliminated by the action of saliva, and the amount of KT that had been retained in the mucosa was determined as described in Section 4.5.1.

The KT extraction retained in the mucosa after the in vitro simulation of the influence of saliva on the elimination of KT was carried out according to Section 4.5.1.

### 4.7. In Vivo Study and Analysis of Tolerance through Histology

With the aim of assessing the tolerability of the alginate-HA gel, the formulation was applied to female pigs (Yorkshire-Landrace) of 45–50 kg. Transmucosal water loss (TMWL) (TEWL-Dermalab) was measured at basal conditions (before applying the formulation) by placing the measuring device perpendicular to the site of interest and reaching a stable TMWL reading in about 60 s. Four pigs were used. Two had the hydrogel applied to the buccal mucosa and two to the sublingual mucosa. The hydrogel was applied on the mucosa covering the most accessible area to facilitate the process and cause the least possible stress to the animals. After two hours, the TMWL value was measured again, and a blood sample was taken to determine whether, by means of buccal and/or sublingual application, therapeutic systemic levels could be reached. Then, all animals were euthanized, and the mucosa tissues were obtained immediately afterwards. The tissues from two treated animals were fixed by immersing them overnight in 4% paraformaldehyde in phosphate-buffered 20 mM, pH 7.4. Then the tissues were processed so as to embed them in paraffin, and vertical histological sections were cut to be stained with hematoxylin and eosin and observed under a Leica DMD 108 optical microscope. The tissues from the two remaining treated animals underwent the extraction procedure for determining the Ketorolac retained in the mucosa, according to Section 4.5.1. The study protocol was approved by the Animal Experimentation Ethics Committee of the University of Barcelona (code 10619, 10 January 2019).

### 4.8. Cytotoxicity

Cell viability was assessed by the MTT (3-(4,5-Dimethylthiazol-2-yl)-2,5-diphenyltetrazolium bromide) assay. This method is based on the mitochondrial reduction of tetrazolium to formazan which is directly proportional to the viable cell numbers. To achieve this, 1 × 104 Caco-2 cells in 100 μL of DMEM medium without phenol red were plated into each well in a 96-well plate and further incubated for five days at 37 °C before the addition of the compounds under study. The volume of each well was set to 0.1 mL, with eight duplicate wells for each specific sample under study. After 24 h, the cells were treated with 0.25% MTT (Sigma-Aldrich) in PBS and allowed to react for 2 h at 37 °C. The supernatant was then removed, and 0.1 mL of dimethyl sulfoxide (DMSO) was added (Applichem, Ecogen, Barcelona, Spain) to each well to fully dissolve the formazan produced by the living cell. For Cell viability determination, the optical density (OD) was measured at a wavelength of 570 nm in a Modulus™ Microplate Photometer (Turner BioSystems, Madrid, Spain). The results were expressed as percentage of cell survival relative to the control (untreated cells).

### 4.9. Analytical Method for the Determination of Ketorolac Tromethamine

To quantify the KT concentration described in previous sections, a HPLC method was used. The chromatographic conditions were the following: the column used was YMC-Pack Pro C18 (25 cm, 4.6 mm and 5 μm). The mobile phase consisted of acetonitrile (+0.065% triethylamine) and purified water (+0.165% acetic glacial acid), in an isocratic elution (1:1) at flux 1 mL/minute. The volume injected was 10 µL, and Ketorolac was determined at the wavelength of 314 nm. The standard range for the calibration line was from 0.39 to 300 μg/mL. Data were collected and processed using Empower Pro software (Walters, Milford, CT, USA).

### 4.10. Statistical Analysis

GraphPad Prism^®^, v. 5.00 software (San Diego, CA, USA), was used for all statistical calculations. If data followed a normal distribution, a *t*-test or an ANOVA (if more than two groups were being compared) was applied. If data followed a non-normal distribution, a Mann-Whitney (for two group comparison) or Kruskal-Wallis test (for more than two groups) was applied. The significance level was 0.05 in all cases.

## 5. Conclusions

The objective of this study was to comprehensively characterize a hydrogel based on sodium alginate and high and low molecular weight hyaluronic acid formulated with 2% ketorolac tromethamine. Organoleptic, morphological, and rheological studies showed the suitability of the formulation to be an excellent and easy topical application of the hydrogel on the mucosa of the oral cavity.

The release studies demonstrated the remarkable capacity of KT to be released from the alginate-HA hydrogel, releasing 51.59% of the drug per cm^2^ in 6 h.

Ex vivo permeation studies demonstrated good oral and sublingual mucosal patency for KT and predicted systemic steady-state concentrations within the therapeutic range. Furthermore, when comparing the results of both mucosae, no statistically significant differences were observed.

An additional positive finding was that comparative studies of the PermeaPad^®^ biomimetic membrane with the buccal and sublingual mucosae showed an excellent correlation but a significantly lower drug retention capacity.

Through in vitro simulation, the influence of saliva on the bioavailability of the drug was observed. It was shown how in one hour, artificial saliva at a constant flow of 0.24 mL/min was capable of eliminating more than half of the initially applied dose on the mucous membranes, which would end up being swallowed and considered as oral administration.

In in vivo studies with pigs and under a finite regime dose, it was not possible to quantify the systemic concentrations of the drug, but the amounts of KT retained in both mucosae showed the feasibility of the gel to provide an analgesic and anti-inflammatory locally, which is very useful in surgical and/or ablative processes such as the elimination of papillomatous lesions, treatment of certain oral carcinomas, dental extractions, etc. Further studies are needed in a multi-dose regimen to characterize the hydrogel’s behavior better, and this would be more realistic since, in practice, a single application of the formulation would not be sufficient to obtain the desired effect.

Finally, the histological, cytotoxicity study and the measured TMWL values demonstrated the safety and innocuousness of the formulation, not showing any damage or alterations to the mucosal tissues.

## Figures and Tables

**Table 1:** Drug release parameters from KT hydrogel according to a first order kinetics. Values represent means ± standard error (n = 3).

Best-Fit Values	PES	PermeaPad^®^
Ymax (%/cm^2^)	51.6 ± 3.2	38.0 ± 6.9
K (h^−1^)	0.66 ± 0.12	0.23 ± 0.14
t^1/2^ (h)	1.05 ± 0.11	3.01 ± 0.27
r^2^	0.98	0.96

Ymax = total amount of drug released; K = release rate constant; t^1/2^ = half time.

**Table 2:** Transmucosal biopharmaceutical permeation parameters of ketorolac 6 h after applying KT hydrogel under an infinite dose regimen according to first-order kinetics, and *p*-values from statistical analysis (one-way ANOVA). Results are expressed as Mean ± SD (n = 3).

Parameter	Buccal Mucosa	Sublingual Mucosa	PermeaPad^®^	*p*-Values
AP (µg/cm^2^)	3790.61 ± 0.24	3864.93 ± 0.25	3041.70 ± 0.67	0.943
AR (µg/cm^2^)	127.34 ± 37.94	150.97 ± 16.13	35.48 ± 13.67	0.003 **
Css (µg/mL)	1.13 ± 0.04	1.21 ± 0.05	1.36 ± 0.14	0.167
Tlag (h)	0.13 ± 0.02	0.38 ± 0.01	0.23 ± 0.04	0.005 **
Jss (µg/h·cm^2^)	666.00 ± 22.74	714.59 ± 27.23	798.80 ± 85.02	0.181
Kp(cm/h)	0.052 ± 0.002	0.056 ± 0.002	0.062 ± 0.007	0.216

** Means statistically significant difference. AP: amount of KT permeated after 6 h. AR amount of KT retained after 6 h. Css: plasma concentration at steady state. Tlag: lag time. Jss: transmucosal/transmembrane flux. Kp: permeability coefficient.

## Data Availability

The data presented in this study are available on request from the corresponding authors. The data are not publicly available due to the fact that they are part of a doctoral thesis, and they will be available once the thesis has been published.

## Abbreviation

The abbreviations used in the manuscript are listed below:

ANOVA	One-way analysis of variance
AP	Amount permeated
AR	Amount retained
C_0_	Initial concentration
CA	Condyloma acuminata
CaCl_2_	Calcium chloride
Clp	Human plasma clearance
CO_2_	Carbon dioxide
Css	Concentration at steady state
d	Diameter
Ext	Extensibility
HPLC	High Performance Liquid Chromatography
HPV	Human Papilloma Virus
J	Flux
Jss	Flux at steady state
K	Release rate constant
KCl	Potassium chloride
KH_2_PO_4_	Potassium dihydrogen phosphate
Kp	Permeability coefficient
KT	Ketorolac tromethamine
Log P	Octanol-water partitioning
NaH_2_PO_4_	Sodium hydrogen phosphate
Na_2_HPO_4_	Disodium phosphate
NaCl	Sodium chloride
NSAID	Non-steroidal anti-inflammatory drug
Pa	Pascals
PBS	Phosphate-buffered saline
PES	Polyethersulfone
r	Coefficient of regression
r^2^	Correlation coefficient
R∞	Maximum amount released
Rt	Amount of drug released at time t
SD	Standard deviation
SEM	Scanning Electron Microscopy
SR	Swelling ratio
t_1/2_	Half-life
TCA	trichloroacetic acid
Tlag	Lag time
TMWL	Trans-mucosal water loss
TSA	Theoretical surface area
Wd	Weight of dried hydrogel
Wi	Initial weigh of hydrogel
WL(%)	Weight loss
Ws	Weight of the swollen hydrogel at different times
Ymax	Total amount of drug released

## Appendix A

Table A1 presents the results of the rheological data fit of alginate-HA 2% KT gel and the goodness of fit for each model.

**Table A1:** Results of rheological data fit in different mathematical models.

Flow Model	R^2^ Ramp-up	Parameters-up	R^2^ Ramp-down	Parameters-down
Newton	0.7401	η = 52.48 Pa∙s	0.6865	η = 48.02
Bingham	0.9248	τ_0_ = 179.5 Pa	0.9727	τ_0_ = 183.3
		η_0_ = 34.76 Pa∙s		η_0_ = 29.64
Ostwald-de Waele	0.9915	K = 200.3	0.9981	K = 164.4
		n = 0.4314		n = 0.4744
Herschel-Bulkley	0.9969	τ_0_ = 151 Pa	0.9999	τ_0_ = 168.4
		K = 345.9		K =313.6
		n = 0.3017		n = 0.3234
Casson	0.9641	τ_0_ = 107.2	0.9887	τ_0_ = 99.31
		η_0_ = 16.32 Pa∙s		η_0_ = 14.54
		n = 0.5		n = 0.5
Cross	0.9999	η_0_ = 326.1 Pa∙s	0.9999	η_0_ = 290.9
		η∞ = 16.41 Pa∙s		η∞ = 0.1397
		$\gamma {˙}_{0}=1.346$		$\gamma {˙}_{0}$= 0.994
		n = 0.6674		n = 0.6876

τ is the shear stress (Pa); η is the dynamic viscosity (mPa·s); $\dot{\gamma }$ is the shear rate (1/s); τ_0_ is the yield shear stress (Pa); η_0_ is the zero-shear rate viscosity; ηp is a constant plastic viscosity (mPa·s); η∞ is the infinity shear rate viscosity; n is the flow index, and K is the consistency index. The goodness of fit was determined by correlation coefficient (r^2^) by linear regression analysis of the flow plots.

Table A2 presents the different mathematical models fitted to the rheological data to characterize the flow curve and the flow properties of alginate-HA 2% KT gel.

**Table A2:** Mathematical models of the flow characterization by regression analysis.

Flow Curve—Models: $\tau =f\left(\dot{\gamma }\right)$	
Newton	$\tau =\eta \cdot \dot{\gamma }$
Bingham	$\tau =\tau_{0}+(\eta_{0}\cdot \dot{\gamma })$
Ostwald-de Waele	$\tau =K\cdot {\dot{\gamma }}^{n}$
Herschel-Bulkley	$\tau =\tau_{0}+K\cdot {\dot{\gamma }}^{n}$
Casson	$\tau =\sqrt[{n}]{\left(\tau_{0}^{n}+{\left(\eta_{0}\cdot \dot{\gamma }\right)}^{n}\right)}$
Cross	$\tau =\dot{\gamma }\cdot (\eta_{\infty }+(\eta_{0}-\eta_{\infty })/(1+{\left(\dot{\gamma }/{\dot{\gamma }}_{0}\right)}^{n})$

τ is the shear stress (Pa); η is the dynamic viscosity (mPa·s); $\dot{\gamma }$ is the shear rate (1/s); *τ*_0_ is the yield shear stress (Pa); *η*_0_ is the zero-shear rate viscosity; *η*p is a constant plastic viscosity (mPa·s); *η*_∞_ is the infinity shear rate viscosity; n is the flow index, and K is the consistency index.

Table A3 describes the calculation of the permeation parameters flux, lag-time, predictive plasma concentration at the steady state and permeability coefficient.

**Table A3:** Permeation Parameters and Calculation of the Css upon Application of alginate-HA gel 2% KT.

Permeation Parameter	Equation	
Steady state plasma concentration	$Css=\frac{J\cdot TSA}{Cl_{p}\cdot A}$	(A1)
Permeability coefficient	$Kp=J\cdot C_{0}$	(A2)

The flux (Jss) corresponds to the slope of the permeation profile’s linear section. The latency time (Tlag) was obtained from the extrapolation of the straight line resulting from the accumulated amounts as a function of time. The steady state plasma concentration (Css) was calculated assuming an area of application of 5 cm^2^ according to the Equation (A1): where Css is the steady state plasma concentration (Css); *J* (µg/h) is the flux; *TSA* (cm^2^) is the theoretical surface area of application; *Cl_p_* (mL/min) is the human plasma clearance of ketorolac, and *A* (cm^2^) is the diffusion area of the Franz cells.

The permeability coefficient was calculated in accordance with the Equation (A2): where *Kp* is the permeability coefficient of ketorolac through the membranes; *J* (µg/h) is the flux, and *C*_0_ (µg/mL) is the initial concentration of ketorolac in the gel.

Table A4 shows the composition of the artificial saliva.

**Table A4:** Artificial saliva composition expressed in concentration (g/L).

Substance Weight	
Pure sodium chloride	8.00 g/L
Potassium dihydrogen phosphate	0.19 g/L
Anhydrous disodium hydrogen phosphate	2.38 g/L
Distilled water	1 L

The resulting pH was 6.8.
